# COVID-19 Vaccine Uptake, Sources of Information and Side Effects Reported by Pregnant Women in Western Australia: Cross-Sectional Cohort Survey

**DOI:** 10.2196/66645

**Published:** 2025-06-23

**Authors:** Nicole Catalano, Shailender Mehta

**Affiliations:** 1 Neonatal Research Team, Fiona Stanley Hospital, 11 Robin Warren Drive, Murdoch, 6150, Australia, 61 0403909285; 2 School of Medicine, Curtin University, Perth, Australia; 3 Neonatal Unit, Fiona Stanley Hospital, Murdoch, Australia; 4 The Kids Research Institute Australia, Perth, Australia

**Keywords:** pregnancy, COVID-19 vaccination, COVID-19, uptake, vaccine uptake, side effects, pregnant, Australia, public health, maternity

## Abstract

**Background:**

Pregnant women are a priority group for COVID-19 vaccination due to their vulnerability as a high-risk cohort. However, the currentCOVID-19 vaccine uptake rate for COVID-19 vaccination among pregnant women in Western Australia remains largely unknown.

**Objective:**

This study aimed to explore pregnant women’s vaccination uptake rates, information sources, and experiences regarding COVID-19 vaccination during pregnancy. We hypothesized that uptake of vaccination among pregnant women is higher than indicated in previous studies, given differences in disease burden and public health restrictions at the time when data was collected.

**Methods:**

A cross-sectional survey was administered electronically to maternity patients at a single tertiary metropolitan hospital in Perth, Western Australia.

**Results:**

A total of 520 women participated in the study. Overall, the antenatal COVID-19 vaccination rate was 79% (n=398). Approximately, 51% (n=256) of the women felt well-informed about the vaccine, and information was sourced primarily from their general practitioner (n=301, 60%), midwives (n=174, 35%), and obstetric doctors (n=64, 13%). Compared to Caucasian women, those of non-Caucasian ethnicity (n=332, 66% vs n=170, 34%; *P*=.07) and those born outside Australia (n=235, 47%) reported lower rates of vaccine information provision by the hospital staff (n=22, 34% vs n=42, 66%; *P*=.04).

**Conclusions:**

The COVID-19 vaccine uptake among pregnant women was encouragingly high in our study, with favorable attitudes and acceptance for the vaccine observed in the majority of pregnant women. This self-reported study also identified opportunities for enhanced cultural competence and further education and training for hospital staff regarding COVID-19 vaccine information provision to ethnically diverse women. Further studies examining such interventions are warranted.

## Introduction

Vaccination against SARS-CoV2 is a key public health strategy to combat the pandemic [1,2]. Pregnant women are a priority group for vaccination against SARS-CoV2, and therefore require education, encouragement, and adequate opportunities to receive vaccinations antenatally [1,3]. Pregnancy puts women in a state of immune deficiency, and therefore increases vulnerability to infection [1], with unvaccinated pregnant women experiencing increased severity of symptoms and higher death rates compared to their vaccinated peers [2,4-6]. Along with increased risk of stillbirth, unvaccinated women also have an increased likelihood of premature birth and neonatal death if they contract SARS-CoV-2 virus during pregnancy [7,8].

At the time of initial vaccination rollout, there was limited evidence regarding the safety of the COVID-19 vaccine in pregnancy [9]. Ongoing real-world studies are adding to the body of evidence, confirming vaccine safety and effectiveness during pregnancy [9-11]. According to advice from the Australian Government and recently completed safety and effectiveness studies, the most widely used mRNA vaccines (eg, Pfizer and Moderna) are safe to be administered throughout pregnancy and the breastfeeding period [1,10].

Worldwide research has demonstrated a hesitancy among women to receive the COVID-19 vaccination during pregnancy, being less likely than any other high-risk cohort to do so [10,12]. Several worldwide studies demonstrate that this uptake remains inadequate, indicating the vaccine acceptance is significantly lower for pregnant women than in nonpregnant women [7,12,13]. An Australian study conducted in 2020 surveyed pregnant women and various maternity health care providers during the early stages of the pandemic and found that pregnant women were the least likely group to accept vaccination [2]. At the time of the study, only 14% of pregnant women had been vaccinated, and 48% of pregnant women indicated they had definite intentions to be vaccinated [2]. Similarly, a large survey questioning vaccine confidence spanning 16 countries reported that only 52% of pregnant women intended to receive the vaccine [10].

Based on worldwide trends, pregnant women are assumed to be an undervaccinated cohort [14]. A 2021 survey of doctors and midwives in Australia reported that 60%‐70% of pregnant women had received their first dose, while an estimated two-thirds had been double vaccinated [15]. A recent study based on data from Western Australia (WA) demonstrated a vaccination uptake rate of only 44% in their pregnant participants [15]. At the time of that study, there was no community transmission of COVID-19, and this was potentially an influencing factor for the surveyed women [15].

There remains a paucity of knowledge regarding Australian pregnant women’s attitudes and their vaccination uptake rate, particularly since the emergence of COVID-19 infections within the Western Australian community and the introduction of proof of vaccination requirements and associated social mandates within WA [15]. Understanding pregnant women’s attitudes toward COVID-19 vaccination may be useful in optimization of vaccination uptake rates, development of educational strategies, and identifying areas for potential interventions such as standing vaccination orders.

## Methods

### Study setting and design

The study’s objective was to determine pregnant women’s perceptions, attitudes, and knowledge regarding vaccination against COVID-19 during pregnancy, and to ascertain the uptake of COVID-19 vaccines during pregnancy within a maternity cohort in a tertiary care setting. We also aimed to explore the effect of various demographic factors on vaccine uptake and vaccine information provision to pregnant women.

This prospective cross-sectional study was conducted at a tertiary metropolitan maternity hospital over a three-month period from January 12, 2022, to April 30, 2022. All pregnant and immediate postpartum women who attended the hospital were eligible to participate in the anonymous survey. Women were approached in the antenatal clinic or the postnatal ward or recruited via a text message containing a link to the optional survey, due to many appointments being transitioned to telehealth as COVID-19 spread across WA. Women who elected to join the study accessed the survey link via a QR code or text link on their own smartphones.

Demographic data collected included gestation and open-text responses for age, ethnicity, country of birth, postal code, and religion. Other multiple-choice questions included education level and sources of information, including various health care providers. The participants were asked at what point during pregnancy information was given, using multiple-choice options for trimesters, and how well-informed they felt, using a Likert scale. In addition to demographic data, the respondents were asked about vaccination status, the number of doses received, the type of vaccine, and the time points in pregnancy when the vaccine was administered. We also gathered information on any side effects using an open-text field for reporting. The unvaccinated participants were invited to describe their reasoning; their responses were a combination of multiple-choice options and a free-text response, allowing women to choose as may answers as desired.

### Statistics

Descriptive statistics were reported using mean and ranges for continuous data and frequency distributions for categorical data. Statistical analysis was performed using *χ*^2^ test for categorical variables. SPSS statistical software (version 24.0; IBM Corp) was used for analysis. P values<.05 were considered statistically significant.

### Ethical Considerations

The study was approved by the South Metropolitan Health Service Human Research Ethics Committee (RGS 5201). Written informed consent was obtained from all participants before commencing the survey. All participants were over 18 years of age and able to provide consent. Data was deidentified upon commencement of the survey, with participants being assigned a unique number. No compensation was provided to the participants.

## Results

A total of 502 eligible women completed the survey in its entirety. Mean age of participants was 31 (SD 5.08; range 18‐46) years.

Overall, 79.2% (n=398) of the pregnant population were vaccinated with at least one dose. Of the study participants, 60.0% (239/398) were double vaccinated and 35.1% (140/398) were triple vaccinated. The majority (328/398, 82.4%) of these women received their vaccinations during pregnancy, while the remaining received them before the pregnancy was identified. A total of 92.4% (368/398) of vaccinated women received the Pfizer, 3.2% (13/398) received AstraZeneca, and 3.2% (13/398) received Moderna vaccines.

Ethnicity was categorized into eight broad groups based on the state maternity database. A majority (332/502, 66.1%) of women identified themselves as Caucasian. Additionally, 2.1% (11/502) identified as Aboriginal, 11.1% (56/502) as Asian, 6.7% (34/502) as Indian, 2.9% (15/502) as Maori, and 2.7% (14/502) as African. An additional 7.9% (n=40/502) women chose to identify as “other” ethnicity. Approximately over half (n=267/502, 53.1%) of the women were born in Australia.

Of the 502 women surveyed, 301 (59.9%) women reported receiving information from their general practitioner (GP) regarding the vaccination, and while the others received it from alternate sources (Table 1). Eighteen percent (89/502) of all participants reported that no one had given them any information about the vaccine, and 5.9% (30/502) of women reported receiving information only in the third trimester of pregnancy.

**Table 1.** Reported sources of vaccine information.

Source of information^a^	Number of respondents (N=502), n (%)
GP	301 (60%)
Midwife	174 (34.6)
Obstetric doctor	64 (12.7)
Other	55 (10.9)
No one	89 (17.7)

a These sources were mutually exclusive.

The GPs provided information to 59.9% (301/502) of all women in our study. Of these, 66.1% were Caucasian and 33.9% were non-Caucasian (odds ratio [OR] 0.961, 95% CI 0.66-1.40; *P=.*85). A total of 174 women reported receiving information from their midwives, and of whom 72.9% (127/174) were Caucasian and 27.1% (47/174) were non-Caucasian (*P*=.22 (OR 1.62, 95% CI 1.08-2.43; *P*=.22). Only 12.7% (64/502) of the women reported receiving information from an obstetric doctor within the hospital system. Of these 64 women, 79.6% (51/64) were Caucasian and 20.3% (13/64) were of minority ethnicity (OR 2.2, 95% CI 1.15-4.15; *P*=.02). Of the 12.7% (64/502) of women who reported receiving information from an obstetric doctor, 65.6% (42/64) were born in Australia and 34.3% (22/64) self-reported as being born elsewhere (OR 1.8, 95% CI 1.04-3.13; *P*=.04).

Sources of information had no significant effect on the uptake rate of the vaccination, whether information was obtained from a GP (OR 1.2, 95% CI 0.78-1.88; *P*=.43), midwife (OR 0.23, 95% CI 0.14-3.6; *P<*.001), obstetric doctor (OR 0.368, 95% CI 0.21-0.64; *P*=.01), other sources (OR 1.59, 95% CI 0.72-3.48; *P*=.29), or from no one (OR 1.63, 95% CI 0.86-3.07; *P*=.15).

Overall, 50.9% (256/502) of women felt well-informed. 39.2% (97/502) women reported feeling somewhat informed, and 9.5% (48/502) reported feeling not well-informed. However, the perceived level of being informed did not statistically affect the uptake rate of the vaccination.

Religion was self-reported by majority of the women and did not have a statistically significant association with vaccine uptake rates (OR 0.9, 95% CI 0.6-1.5; *P*≥.99).

The majority of women (194/398, 48.7%) received two doses of the vaccine in the second trimester of pregnancy. Table 2 shows the side effects profile reported by the participants. Of the vaccinated women, 63.3% (252/398) reported experiencing some side effects from the vaccine, most commonly fatigue (n=81, 55%)), sore arm (n=65, 44%), headaches (n=51, 35%), body aches (n=39, 26%), fever (n=31, 21%), and nausea or vomiting (n=24, 16%). There was no difference in the frequency or type of side effects among women under or over 30 years, (OR 1.14, CI 95% 0.75-1.75; *P*=.59] or across various ethnic groups: Caucasian (n=334, 67%); Aboriginal (n=11, 2%); Asian (n=56, 11%); Indian (n=34, 7%); Maori (n=15, 3%); African (n=14, 3%); Polynesian (n=2, 0.4%); Middle Eastern (n=4, 1%), and other ethnic groups (n=31, 6%) *(P*=.82).

Among the unvaccinated women, 405 reasons were cited. “Safety concerns” was the main reason for women opting not to receive the vaccination (137/405, 33.8%). Additionally, 21.2% (n=86) of the women were concerned about adverse effects on the baby, 12.5% (n=51) were concerned about effects on themselves, while 16.0% (65/405) opted to wait until after their baby was born (Table 3).

**Table 2.** Vaccination side effects profile reported by participants.

Vaccination side effects^a^	Number of respondents (N=252), n (%)
Fatigue	81 (55)
Sore Arm	65 (44)
Headaches	51 (35)
Fever	31 (21)
Nausea/Vomiting	24 (16)

a The side effects were not mutually exclusive.

**Table 3.** Reasons for women not receiving the vaccine.

Reasons for not receiving vaccine^a^	Number of respondents (n=405), n (%)
Adverse effect on myself	51 (12.5)
Adverse effect on baby	86 (21.2)
I am waiting until after baby is born	65 (16.0)
No immediate COVID-19 threat in WA^b^	14 (3.4)
Upcoming booking	23 (5.6)
Not getting the vaccine	14 (3.4)
Other	8 (1.5)

a These effects are not mutually exclusive.

b WA: Western Australia.

The respondents’ education levels varied widely: high-school diploma (n=172, 35%); a postgraduate degree (n=224, 45%); a master’s degree (n=53, 11%); a trade qualification (n=31, 61%); a doctorate ( n=8, 2%); and no education (n=10, 2%). The level of education had no significant impact on the vaccine uptake rates *(P*>.05).

## Discussion

Overall, the rate of the COVID-19 vaccination within our study’s pregnant population was encouragingly high, although it lagged behind the state average of >95% double vaccination in the general population [15]. The diverse demographic of the cohort at our hospital reportedly had an equal uptake of the vaccination regardless of age, ethnicity, education level, or country of birth. To our knowledge, this is among the highest vaccination rates in a pregnant population globally, although a deficit of research on this topic remains [6,12,15-17].

Every two out of three women received COVID-19 vaccine information from their GPs. Although this number is higher than that of women receiving vaccine information from the hospital staff, given the context of a pandemic and pregnancy being a vulnerability, it would be reasonable to aim for all women to receive information on COVID-19 vaccine during their first consultation with GPs, midwives, or obstetric doctors. Workload pressures on GP clinics and hospitals during the pandemic undoubtedly placed strain on service and information delivery but also highlighted missed opportunities to prioritize vaccine information at the earliest opportunity. The pandemic may also have affected the ability of pregnant women to have timely appointments with their GPs due to restrictions and increased burden on practices [18,19]. In our study, 10% (n=48) of women reported that they did not feel well-informed about the vaccination, reiterating the need to educate pregnant women. Some women (n=30, 6%) reported receiving information for the first time only in the third trimester of pregnancy.

Eighteen percent (n=89) of women reported not receiving any information at all, which is a public health concern. These data were not influenced by age, ethnicity, or religion of the women. Encouraging multiple open conversations surrounding vaccination between all health care providers and pregnant women may possibly decrease the number of women who reported not receiving any information [20].

Australia’s historically strong population growth is drawn from both natural increase and net overseas migration. In 2020, there were over 7.6 million migrants living in Australia, accounting for 29.8% of the population who were born overseas [21]. It was reassuring to see that there was no difference in responses on receiving information from GPs across various ethnic groups or countries of birth. However, the significantly high proportion of women from minor ethnic groups or those born outside Australia who reported receiving no information from the hospital staff is highly concerning. Potentially, language barriers, inadequate use of interpreters, or lack of cultural competence may contribute to this number in our culturally diverse study population; however, these factors were not investigated further in our study. Ensuring a diversified workforce in hospitals may benefit women through the dispersion of information from culturally diverse health care professionals.

A recent, similar study conducted within WA by Ward et al [22] explored women’s vaccine uptake rate and attitudes surrounding vaccination. While similar information was obtained from the pregnant population, the results contrasted significantly between the two studies; this could potentially be attributed to differences in community transmission as well as the vaccination-related social restrictions and introduction of state government vaccine mandates within WA. The uptake rate in the aforementioned study was only 44%, compared to 79% in our study. Additionally, one-third of their study population reported not being given any information regarding the vaccine, whereas our numbers, although still concerning, were much lower at 18%. This difference may likely be attributed to increased awareness and education as the pandemic evolved in WA, along with real-time evidence of vaccine safety in pregnancy being reported worldwide. At the commencement of our study, there was no community spread of COVID-19, and therefore, no imminent urgency was experienced within WA. However, the opening of borders was announced soon after the vaccine mandates were introduced, and eventually, the interstate and international WA borders were opened during the course of our study. Both studies discovered that women’s safety concerns regarding their baby and themselves were primary factors behind declining or delaying the vaccine administration.

Our study had various strengths and limitations. It was conducted throughout the community COVID-19 transmission in WA, making our findings unique and relevant to the current pandemic situation. Our study population reflects the health status and demographic status of WA’s pregnant population. It incorporated multiple ethnicities, wide age range, and both low- and high-risk pregnancies. The results are generalizable to the WA pregnant population, as a variety of ethnicities, education levels, and ages were incorporated, along with a large sample size. The limitations of our study include restriction to a single-center experience, lack of information on primary spoken languages by the women, and reliability on the self-reported responses by the pregnant women.

In conclusion, COVID-19 vaccination was widely accepted among our study population, in contrast to previously reported data. Western Australia has demonstrated a high uptake of the vaccination across all age, social, religious, and ethnic groups attending our center. There were minimally reported and mild side effects due to the vaccine. In the exploration of women’s attitudes, safety concerns led to vaccine hesitancy. Optimizing the uptake rate remains a priority for this vulnerable cohort to improve maternal and neonatal outcomes. The information gained from this study can be used to encourage hesitant women by providing the positive data. The discrepancy in information provided between the groups may indicate a need to address cultural competency among staff, furthering staff education, and ensuring a diverse workforce. Further studies examining such interventions are warranted to confirm the influence of these strategies on the vaccine uptake among pregnant women.

## Abbreviations

GP: general practitioner
WA: Western Australia
